# Association of Clinical Phenotypes in Haploinsufficiency A20 (HA20) With Disrupted Domains of A20

**DOI:** 10.3389/fimmu.2020.574992

**Published:** 2020-09-23

**Authors:** Yu Chen, Zhenghao Ye, Liping Chen, Tingting Qin, Ursula Seidler, De'an Tian, Fang Xiao

**Affiliations:** ^1^Department of Gastroenterology, Tongji Hospital of Tongji Medical College, Huazhong University of Science and Technology, Wuhan, China; ^2^Department of Biliary–Pancreatic Surgery, Tongji Hospital, Tongji Medical College, Huazhong University of Science and Technology, Wuhan, China; ^3^Department of Gastroenterology of Hannover Medical School, Hanover, Germany

**Keywords:** haploinsufficiency A20, autoinflammatory disorders, monogenic disease, TNFAIP3, OTU domain, ZnF domain, clinical manifestation

## Abstract

**Background:** Haploinsufficiency A20 (HA20) is a newly described monogenic disease characterized by a wide spectrum of manifestations and caused by heterozygous mutations in *TNFAIP3* which encodes A20 protein. *TNFAIP3* mutation leads to disruption of the A20 ovarian tumor (OTU) domain and/or the zinc finger (ZnF) domain. This study aims at exploring the association between the various manifestations of HA20 and different domains disruption of A20.

**Methods:** We reviewed the HA20 cases in previous literature and summarized the clinical features, *TNFAIP3* mutation loci and the disrupted domains caused by different sites and patterns of mutations. Patients were classified into three groups according to the A20 domains disruption.

**Results:** A total of 89 patients from 39 families with a genetic diagnosis of HA20 were included. Overall, the age at onset of HA20 was early (median:5.92, IQR:1-10). Patients in the ZnF group showed the earliest onset (median:2.5, IQR:0.6-5), followed by patients in the OTU+ZnF group (median:6, IQR:1-10) and patients in the OTU group (median:10, IQR:8-14). The main manifestations of HA20 patients were recurrent oral ulcers (70%), recurrent fever (42%), gastrointestinal ulcers (40%), skin lesion (38%), genital ulcers (36%), and musculoskeletal disorders (34%). The percentage of patients with musculoskeletal disorders was significantly different among the three groups (*p* = 0.005). Patients in the OTU+ZnF group and ZnF group were more likely to develop musculoskeletal disorders than patients in the OTU group (*p* = 0.002 and *p* = 0.035, respectively). Besides, forty-three percent of HA20 patients were initially diagnosed as Behcet's disease (BD). Compared to the ZnF group, the OTU+ZnF group and OTU group had a higher percentage of patients initially diagnosed as BD (*p* = 0.006 and *p* < 0.001, respectively).

**Conclusion:** HA20 is characterized by early-onset and the most common symptoms of HA20 are recurrent oral ulcers, fever and gastrointestinal ulcers. The onset of HA20 in patients with the ZnF domain disruption is earlier than patients with the OTU domain disruption. Compared to the OTU domain, the ZnF domain may be more closely related to musculoskeletal disorders.

## Introduction

A20, a protein encoded by the tumor necrosis factor alpha-induced protein 3 gene (*TNFAIP3*), is a crucial negative regulator of inflammation (1, 2). It is well- known to inhibit NF-κB signaling and has recently been shown to restrict the interferon regulatory factor (IRF) pathway and autophagy (3). A20 consists of two distinct domains: an amino-terminal ovarian tumor (OTU) domain and a carboxy-terminal zinc finger (ZnF) domain (4). The ZnF domain contains K63-linked E3 ubiquitin ligase and polyubiquitin-binding ability, while the OTU domain carries deubiquitinating activity (1). Different *TNFAIP3* mutations may result in distinct A20 domain disruption and symptomatic manifestation depending on their type and location.

Haploinsufficiency A20 (HA20) is a monogenic disease caused by heterozygous *TNFAIP3* mutations and is characterized by inflammation in multiple organs (5). Since the first case was reported in 2016 (5), more attention has been paid to HA20 and an increased number of cases has been reported. The manifestations of patients with HA20 are complicated and show individual variation (6, 7). It is assumed that the diverse clinical manifestations of HA20 may result from the variable expressivity in the autosomal-dominant inheritance pattern, the interaction with other genes and environments influences (8). Previous mouse models indicate that disruption of different A20 domains may result in different clinical manifestations of HA20. For example, HA20 symptoms in mice with the disrupted ZnF domain (A20^ZnF7/ZnF7^ mice, A20^ZnF4ZnF7/ZnF4ZnF7^ mice) presented differently than mice with the disrupted OTU domain (A20^OTU/OTU^ mice) (2, 9). However, it remains uncertain whether different forms of A20 domain disruption directly affect the variable manifestations of HA20 patients. Here, we analyze the relationship between A20 domain disruption and corresponding clinical characteristics of HA20 patients.

## Methods

### Literature Search Strategy

We searched literature up to March 2020 that reported HA20 patients. The search was conducted using PubMed, Google scholar, and the Chinese database CNKI. The keywords “haploinsufficiency A20” AND “autoinflammation,” or “*TNFAIP3* mutation” AND “autoinflammation” were used for the search. No language or publication date restrictions were applied to the search.

### Inclusion and Exclusion Criteria

The inclusion criteria for eligible cases were that the HA20 patients should be reported with the *TNFAIP3* gene mutation by genetic analysis. Patients with additional gene mutations were excluded.

### Data Collection

The following information was extracted from each eligible HA20 case: age of HA20 onset, gender, details of *TNFAIP3* gene mutation, initial diagnosis, clinical manifestations, treatment, and response to treatment. We found a total of 33 *TNFAIP3* mutations at various loci (5, 8, 10–12). These mutations could result in OTU and/or ZnF domain disruption and were identified as missense, nonsense or frameshift mutations. Patients were divided into three groups according to the disrupted domain caused by *TNFAIP3* mutation: (1) nonsense and frameshift mutations in the OTU coding region that impaired the function of both OTU and ZnF domains (OTU+ZnF group), (2) missense mutations in OTU coding region that disrupted the OTU domain only (OTU group), and (3) mutations in the ZnF coding region that disrupted the ZnF domain function (ZnF group). The age of onset for HA20 patients was defined as the age when the initial disease symptoms were reported. The term “patients responded to treatment” in our study refers to the reported improvement of symptoms after treatment. To analyze the correlation between specific *TNFAIP3* mutations and the severity of symptoms in HA20, we classified H20 symptoms severity into three categories based on the number of organs involved: (1) mild: <4 organs involved, (2) moderate: 4 to 6 organs involved, (3) severe: more than 6 organs involved.

### Statistical Analysis

All statistical analyses were performed with SPSS 19 (IBM, New York, USA) software. The age of HA20 onset was processed as median values with interquartile ranges (IQR). Group differences in the age of onset were compared by the Kruskal-Wallis test. Chi-square tests were used to evaluate differences between two or three groups and Fisher's exact test was used when the theoretical frequency was <5. A two-sided *p* < 0.05 indicated statistical significance.

## Results

### Classification of A20 Disruption According to *TNFAIP3* Gene Mutations

**Figure 1:**
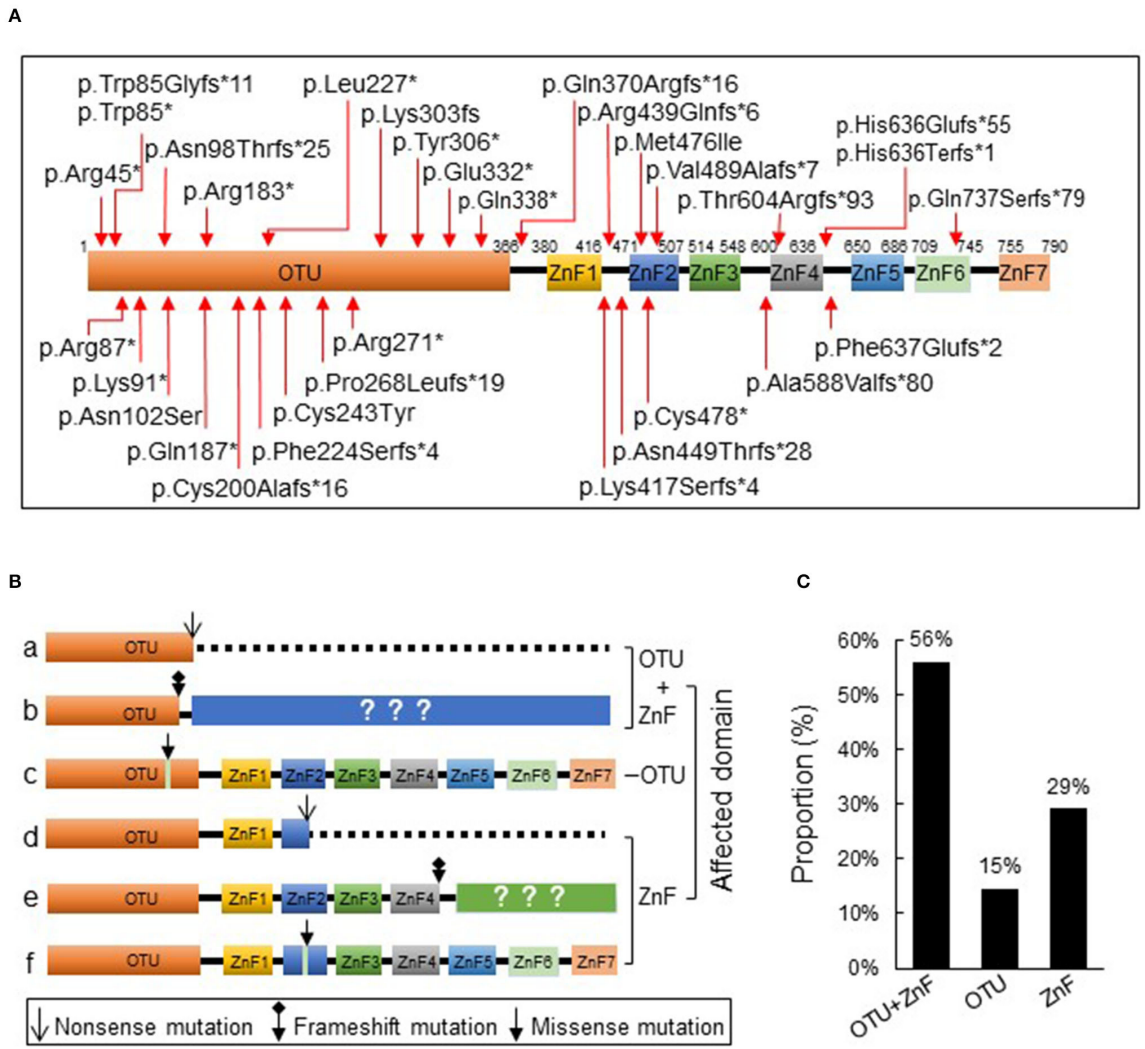
Schematic diagram of A20 protein and its three types of disruption. **(A)** The protein A20 consists of an ovarian tumor (OTU) domain and seven zink-finger (ZnF) domains. The reported TNFAIP3 mutations from literature are indicated with arrow. **(B)** The reported TNAFIP3 mutations can result in three types of A20 disruption: disruption of both OTU and ZnF functional domains (a-b, OTU+ZnF), disruption of single functional domain OTU (c, OTU) or ZnF (d-f, ZnF). **(C)** The proportion of patients in each group. Patients were classified into the OTU+ZnF group, the OTU group and the ZnF group according to three types of A20 disruption.

We initially collected a total of 93 cases according to the inclusion criteria and 4 patients were excluded from the study due to the presence of additional mutations. The remaining 89 patients from 39 families were reported with heterozygous loss-of-function mutations in *TNFAIP3* (Table S1) which showed evident familial aggregation. We identified 33 disease-causing variants and 32 of which were indicated in Figure 1A. A large deletion mutation of exons 2–3 of *TNFAIP3* located in the OTU coding region was not shown on the schematic diagram because no exact mutation locus was identified in the case report (13). We found 7 frameshift mutations and 11 nonsense mutations in the OTU coding region (OTU+ZnF group), 2 missense mutations in the OTU coding region (OTU group), 11 frameshift mutations, 1 nonsense, and 1 missense mutation in the ZnF coding region (ZnF group) (Figure 1B). There were 50 patients (56%) from 23 families in the OTU+ZnF group, 13 patients (15%) from 3 families in the OTU group and 26 patients (29%) from 3 families in the ZnF group, respectively (Figure 1C, Table S1).

### Clinical Characteristics of Patients With HA20

**Figure 2:**
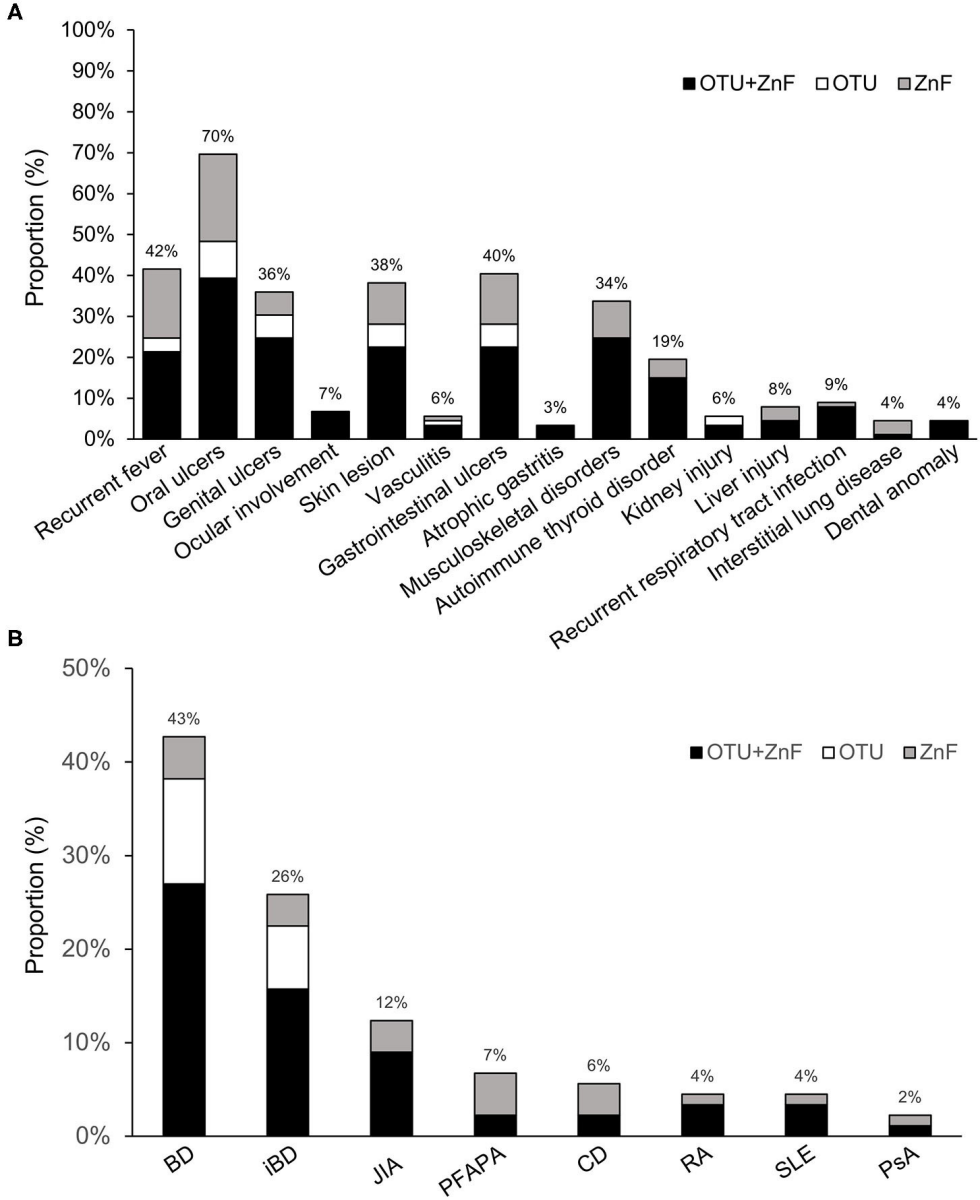
Statistics of clinical manifestation in patients with HA20. **(A)** The percentage of various symptoms of HA20 in 89 patients. Patients were classified into the OTU+ZnF group, the OTU group and the ZnF group according to three types of A20 disruption. Except for interstitial lung disease, the patients with specific symptom of HA20 were mainly from OTU+ZnF group. **(B)** The proportion of initial diagnosis in HA20 and the distribution of each diagnosis in three groups.

We observed diverse clinical manifestations in the 89 HA20 patients evaluated for this study. Overall, the most common symptom was oral ulcers (70%), followed by recurrent fever (42%), gastrointestinal ulcers (40%), skin lesions (38%), genital ulcers (36%), musculoskeletal disorders (34%), and autoimmune thyroid disorder (19%). Less than 10% of HA20 patients presented with ocular involvement (7%), vasculitis (6%), atrophic gastritis (3%), kidney injury (6%), liver injury (8%), recurrent respiratory tract infection (9%), interstitial lung disease (4%), or dental anomaly (4%) was. Additionally, while the majority of patients with interstitial lung disease were in the ZnF group, patients with specific symptoms were mainly distributed in the OTU+ZnF group (Figure 2A).

The initial diagnoses of HA20 patients in each of the three groups were shown in Figure 2B. Eighty percent (72/89) of patients were initially diagnosed with autoimmune diseases other than HA20. Forty-three percent of patients were initially diagnosed with Behcet's disease (BD), an autoimmune disease that cause vascular inflammation throughout the body, representing the largest proportion of initial diagnoses in HA20 patients. Twenty-six percent of HA20 patients met the diagnostic criteria of intestinal BD (iBD), with the hallmark features of BD and gastrointestinal ulcers (14). Other initial diagnoses of HA20 included juvenile idiopathic arthritis (JIA) (12%), periodic fever with aphthous pharyngitis and adenitis (PFAPA) (7%), Crohn's disease (CD) (6%), rheumatoid arthritis (RA) (4%), systemic lupus erythematosus (SLE) (4%), and psoriatic arthritis (PsA) (2%). Patients who were initially diagnosed with BD, iBD, JIA, RA, or SLE were mainly a part of the OTU+ZnF group. No patients in the OTU group were initially diagnosed with JIA, PFAPA, CD, RA, SLE, PsA.

### Comparison of Clinical Characteristics According to Types of A20 Disruption

Some symptoms of HA20 exhibited significantly different occurrences among the three groups. Patients with only the OTU domain or ZnF domain disruption did not present with ocular dysfunction, atrophic gastritis, or dental anomalies. Patients in the OTU group did not present with musculoskeletal disorders, autoimmune thyroid disorder, liver injury, recurrent respiratory tract infections, or interstitial lung disease. No patients in the ZnF group developed kidney injury.

The gender distributions were similar among patients in OTU+ZnF, OTU, and ZnF groups. The overall age of HA20 onset was early (median: 5.92, IQR:1–10), however there was a significant difference in age onset between the three groups (*p* < 0.001). Compared to patients in the OTU group (median onset age:10, IQR: 8–14), patients in the OTU+ZnF group (median onset age: 6, IQR: 1.165–7) and the ZnF group (median onset age: 2.5, IQR: 0.6–5) showed earlier onset (all, *p* < 0.001). Patients in the ZnF group showed earlier onset of HA20 than patients in the OTU+ZnF group (*p* = 0.040) (Table 1). We further analyzed the specific loci of *TNFAIP3* mutations in these patients to identify which mutations may confer the earliest HA20 onset. We found that patients with the p.Leu227^*^ mutation in the OTU+ZnF group appeared to confer the earliest onset of HA20 (median onset age:0.83, IQR: 0–1.25) (Figure S1).

**Table 1:** Univariate analysis of clinical characteristics in patients with HA20 based on types of A20 disruption.

**Clinical Feature**	**Total (*n* = 89)**	**OTU+ZnF (*n* = 50)**	**OTU (*n* = 13)**	**ZnF (*n* = 26)**	**χ2/Fisher**	***p* value**
**Age of onset(y) median (IQR)**	5.92 (1–10)	6 (1.165-7)	10 (8–14)	2.5 (0.6-5)	18.269	<0.001
						<0.001^a^
						0.040^b^
						<0.001^c^
**Gender**					1.615	0.453
Male	40% (36/89)	38% (19/50)	31% (4/13)	50% (13/26)		
Female	60% (53/89)	62% (31/50)	69% (9/13)	50% (13/26)		
**Manifestation**						
Recurrent fever	42% (37/89)	38% (19/50)	23% (3/13)	58% (15/26)	4.707	0.096
Oral ulcers	70% (62/89)	70% (35/50)	62% (8/13)	73% (19/26)	0.552	0.759
Ocular involvement	7% (6/89)	12% (6/50)	0% (0/13)	0% (0/26)	3.810	0.109
Genital ulcers	36% (32/89)	44% (22/50)	38% (5/13)	19% (5/26)	4.600	0.100
Skin lesion	38% (34/89)	40% (20/50)	38% (5/13)	35% (9/26)	0.211	0.900
Gastrointestinal ulcers	40% (36/89)	40% (20/50)	38% (5/13)	42% (11/26)	0.063	0.969
Vasculitis	6% (5/89)	6% (3/50)	8% (1/13)	4% (1/26)	0.622	1
Atrophic gastritis	3% (3/89)	6% (3/50)	0% (0/13)	0% (0/26)	1.440	0.720
Musculoskeletal disorders	34% (30/89)	44% (22/50)	0% (0/13)	31% (8/26)	10.239	0.005
						0.002^a^
						0.327^b^
						0.035^c^
Autoimmune thyroid disorder	19% (17/89)	26% (13/50)	0% (0/13)	15% (4/26)	4.736	0.089
Kidney injury	6% (5/89)	6% (3/50)	15% (2/13)	0% (0/26)	3.555	0.118
Liver injury	8% (7/89)	8% (4/50)	0% (0/13)	12% (3/26)	1.213	0.559
Recurrent respiratory tract infection	9% (8/89)	14% (7/50)	0% (0/13)	4% (1/26)	2.691	0.233
Interstitial lung disease	4% (4/89)	2% (1/50)	0% (0/13)	12% (3/26)	3.240	0.173
Dental anomaly	4% (4/89)	8% (4/50)	0% (0/13)	0% (0/26)	2.097	0.354

a *Comparison of the clinical manifestations between OTU+ZnF and OTU groups*.

b *Comparison of the clinical manifestations between OTU+ZnF and ZnF groups*.

c *Comparison of the clinical manifestations between OTU and ZnF groups*.

The incidence of musculoskeletal disorders in HA20 patients was significantly different among the three patient groups (*p* = 0.005). No patients in the OTU group developed musculoskeletal disorders (0%). However, patients in the OTU+ZnF (44%) and ZnF groups (31%) were more likely to develop musculoskeletal disorders (*p* = 0.002 and *p* = 0.035, respectively), with no significant difference between the two groups (*p* = 0.327) (Table 1). Next, we explored which *TNFAIP3* mutation loci correlated with more severe symptoms (Table S2). We found that patients with the p.Phe224Serfs^*^4 mutation were more likely to show severe symptoms (Table S2). Patients with the p.Leu227^*^ or p.Gln370Argfs^*^16 mutations were more likely to show moderate to severe symptoms (Table S2).

Most patients experienced improved symptoms after immunomodulatory treatment. Twenty-two patients were treated with biological agents, 19 of which responded well to the therapy (response rate: 86%). The response rates of glucocorticoid treatments, disease-modifying antirheumatic drugs, and colchicine were 71, 70, and 45%, respectively (Table S2). Two patients improved after autologous hematopoietic stem cell transplantation. However, treatment response in HA20 patients were variable. The response rate of colchicine was significantly different among the three patient groups (*p* = 0.009). Patients in the OTU+ZnF group were more likely to respond to colchicine treatment compared with patients in the OTU group (*p* = 0.005). The response rate of other treatments was comparable across the three groups (Table 3).

### Comparison of Initial Diagnosis in HA20 Patients According to Types of A20 Disruption

The percentage of HA20 patients who were initially diagnosed with BD was significantly different among patients in the three groups (*p* = 0.001). The proportion of patients initially diagnosed with BD in the OTU+ZnF (48%) and OTU (77%) groups was significantly higher than the ZnF group (15%) (*p* = 0.006 and *p* < 0.001, respectively), with no significant difference between the OTU+ZnF and the OTU groups (*p* = 0.116). We observed no significant difference in initial diagnosis of iBD, JIA, PFAPA, CD, RA, SLE, or PsA between the three patient groups (Table 2).

**Table 2:** Univariate analysis of initial diagnosis in HA20 patients based on types of A20 disruption.

**Initial diagnosis of HA20**	**Total (*n* = 89)**	**OTU+ZnF (*n* = 50)**	**OTU (*n* = 13)**	**ZnF (*n* = 26)**	**Fisher**	***P*-value**
BD	43% (38/89)	48% (24/50)	77% (10/13)	15% (4/26)	15.044	0.001
						0.116^a^
						0.006^b^
						<0.001^c^
iBD	26% (23/89)	28% (14/50)	46% (6/13)	12% (3/26)	5.654	0.068
JIA	12% (11/89)	16% (8/50)	0% (0/13)	12% (3/26)	2.052	0.362
PFAPA	7% (6/89)	4% (2/50)	0% (0/13)	15% (4/26)	3.526	0.172
CD	6% (5/89)	4% (2/50)	0% (0/13)	12% (3/26)	2.087	0.418
RA	4% (4/89)	6% (3/50)	0% (0/13)	4% (1/26)	0.508	1.000
SLE	4% (4/89)	6% (3/50)	0% (0/13)	4% (1/26)	0.508	1.000
PsA	2% (2/89)	2% (1/50)	0% (0/13)	4% (1/26)	0.920	1.000

a *Comparison of the initial diagnosis between OTU+ZnF and OTU groups*;

b *Comparison of the initial diagnosis between OTU+ZnF and ZnF groups*;

c *Comparison of the initial diagnosis between OTU and ZnF groups. BD, Behcet's disease; iBD, intestinal Behcet's disease; JIA, juvenile idiopathic arthritis; PFAPA, periodic fever with aphthous pharyngitis and adenitis; CD, Crohn's disease; RA, rheumatoid arthritis; SLE, systemic lupus erythematosus; PsA, psoriatic arthritis*.

**Table 3:** Univariate analysis of treatment response of patients with HA20 based on types of A20 disruption.

**Treatments**	**Total**	**OTU+ZnF**	**OTU**	**ZnF**	**χ2/ Fisher**	***P* value**
Biological agents	86%(19/22)	94%(15/16)	(0/0)	67%(4/6)	-	0.169
Glucocorticoid	71%(20/28)	69(11/16)	100%(4/4)	63%(5/8)	1.681	0.532
DMARDs	70%(14/20)	67%(10/15)	100%(1/1)	75%(3/4)	0.690	1.000
Immuno-suppressants	65%(11/17)	75%(9/12)	0%(0/1)	50%(2/4)	2.716	0.344
Colchicine	45%(13/29)	67%(10/15)	0%(0/7)	43%(3/7)	8.826	0.009
						0.005^a^
						0.376^b^
						0.192^c^

a *Comparison of the treatment response between OTU+ZnF and OTU groups*;

b *Comparison of the treatment response between OTU+ZnF and ZnF groups*;

c *Comparison of the treatment response between OTU and ZnF groups. DMARDs, disease-modifying antirheumatic drugs*.

## Discussion

HA20 is an autosomal-dominant-inherited disease characterized by systemic inflammation in multiple organs with a wide spectrum of manifestations (3). The specific mechanisms of this individual clinical variation in HA20 patients remain poorly understood. Here, we summarize the clinical characteristics of HA20 and highlight that distinct domain disruption of A20 influences the variation in HA20 symptoms and onset among patients.

Early HA20 recognition and diagnosis remain challenging due to its diverse clinical manifestations. The 89 cases of HA20 we evaluated featured a wide variety of symptoms including recurrent fever, oral ulcers, genital ulcers, ocular disorders, skin lesion, vasculitis, gastrointestinal ulcers, atrophic gastritis, musculoskeletal disorders, autoimmune thyroid disorder, kidney injury, liver injury, recurrent respiratory tract infection, interstitial lung disease, and dental anomalies. Based on these symptoms, some patients were initially diagnosed with autoimmune diseases other than HA20, such as BD or JIA. Consistent with previous reports (6, 7), we found that the hallmark features of BD, including oral ulcers, genital ulcers, gastrointestinal ulcers, and skin lesions were also frequent in HA20 patients. Therefore, patients with these symptoms were more likely to be initially diagnosed as BD (43%) than other autoimmune diseases (2–12%). Most HA20 patients exhibited early-onset of symptoms (median: 5.92, IQR: 1–10), which may aid proper diagnosis of HA20 over other autoimmune diseases and promote early HA20 diagnosis.

Our study suggests that mutations in different domains of the A20 protein might influence a variety of specific HA20-related manifestations in a domain-dependent manner. We divided the 89 HA20 patients into an OTU+ZnF group (50/89), an OTU group (13/89), and a ZnF group (26/89) according to the particular A20 domains that was disrupted. Notably, patients with the disrupted ZnF domain in either the OTU+ZnF or ZnF group presented symptoms earlier than patients with only the disrupted OTU domain. The early median overall onset of HA20 suggests that both the OTU and ZnF domains may impact the early onset of HA20. The earlier median age of onset in patients with the disrupted ZnF domain implies that the ZnF domain might play a more vital role in the early HA20 onset. Recent evidence suggests that the A20 protein restricts inflammation through E3 ligase activity and the ubiquitin-binding activity of the ZnF domain (9, 15). Prior studies found that A20^ZnF7/ZnF7^ mice presented arthritis or physical retardation by 9 weeks in age, and A20^ZnF4ZnF7/ZnF4ZnF7^ mice developed inflammation in multiple organs within 3 weeks of birth (2, 9, 15). Comparatively, A20^OTU/OTU^ mice gradually developed splenomegaly at 6 months of age with increased myeloid cells (16, 17). These findings indicated that mice with the disrupted ZnF domain (A20^ZnF7/ZnF7^ mice, A20^ZnF4ZnF7/ZnF4ZnF7^ mice) developed severe pathological signs earlier than mice with the disrupted OTU domain (A20^OTU/OTU^ mice). The earlier age of onset in mice with the disrupted ZnF domain supported the implication that the ZnF domain might be more relevant to the early onset of HA20.

Here, we show that patients with disrupted OTU plus ZnF domains or just the ZnF domain were more likely to develop musculoskeletal disorders than patients with only the disrupted OTU domain. Additionally, the incidence of musculoskeletal disorders was not significantly different between the OTU+ZnF group and the ZnF group. These results suggest that the ZnF domain may play an important role in the pathogenesis of musculoskeletal disorders in HA20 patients. Further, mice with the dysfunctional ZnF domain showed spontaneous arthritis with increased Th17 cells and higher production of proinflammatory cytokines than control mice (15). The ZnF domain was also reported to prevent inflammasome-dependent arthritis by inhibiting macrophage necroptosis through its ubiquitin-binding domain (2). We propose that the ZnF domain of A20 might influence musculoskeletal disorders more than the OTU domain in H20.

In our study, patients in the OTU+ZnF and OTU groups were more likely to be initially diagnosed with BD compared to patients in the ZnF group. One potential explanation for this is that they were more likely to present BD-like symptoms, including recurrent oral ulcers, genital ulcers, gastrointestinal ulcers, skin lesions, and ocular lesions (18).

There were several limitations in our study. Since HA20 is a newly identified rare monogenetic disease, the study was limited by the sample size. Additionally, patients with disruption of ZnF (1–7) domains should be further classified into subgroups to increase understanding of ZnF's mechanistic role in HA20. Finally, the recall bias of the patient's history may have influenced the analysis of HA20 patients' manifestations.

In conclusion, our findings suggest that inflammatory lesions of HA20 generally start in early childhood and the most common symptoms are recurrent oral ulcers, fever, and gastrointestinal ulcers. Compared to patients with disruption in the OTU domain, patients with the ZnF domain disruption tend to show earlier onset of HA20 with a higher risk of musculoskeletal disorders. Further work should explore the specific molecular mechanisms of these different A20 domains that affect the manifestations of HA20.

## Data Availability Statement

The raw data supporting the conclusions of this article will be made available by the authors, without undue reservation.

## Author Contributions

FX and YC: study concept and design, acquisition, analysis and interpretation of data, and drafting of the manuscript. ZY, LC, and TQ: collection, analysis and interpretation of data. US and DT: critical review of the manuscript. All authors contributed to the article and approved the submitted version.

## Conflict of Interest

The authors declare that the research was conducted in the absence of any commercial or financial relationships that could be construed as a potential conflict of interest.
