# Supplementary material for: Association of Clinical Phenotypes in Haploinsufficiency A20 (HA20) With Disrupted Domains of A20
Source: Front Immunol. 2020 Sep 23;11:574992. doi: 10.3389/fimmu.2020.574992 (PMC7546856; doi:10.3389/fimmu.2020.574992)
Supplement: Supplementary file 1 [file Data_Sheet_1.PDF]

Supplementary Material

Figure S1

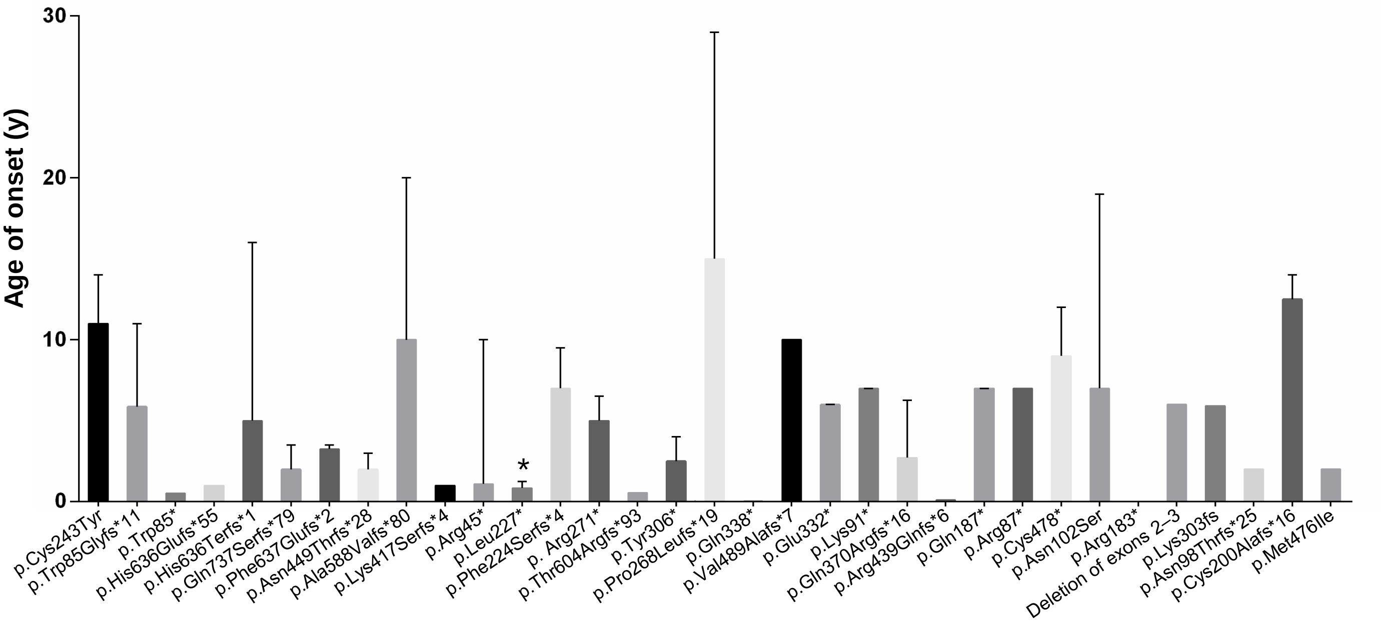

Figure S1. The onset age of patients with specific mutation loci. The differences in the onset age of patients with various mutation loci were compared by the Kruskal-Wallis test. Patients with the p.Leu227* mutation showed significantly early onset of HA20 (median:0.83, IQR:0-1.25). Data were presented as median ± IQR.
