# Supplementary material for: Association of Clinical Phenotypes in Haploinsufficiency A20 (HA20) With Disrupted Domains of A20
Source: Front Immunol. 2020 Sep 23;11:574992. doi: 10.3389/fimmu.2020.574992 (PMC7546856; doi:10.3389/fimmu.2020.574992)
Supplement: Supplementary file 2 [file Data_Sheet_2.PDF]

***Supplementary Material***

Table S1. Clinical characteristics of patients with HA20.

| Family (Ref.) | Amino acid alteration | Patient | AOS (y) | Gender | RF | Oral ulcers | Genital ulcers | Ocular | Skin lesion | Vasculitis | GI ulcers | MSD | AITD | RRTI | Other manifestations | Initial diagnosis | Treatment |
|---|---|---|---|---|---|---|---|---|---|---|---|---|---|---|---|---|---|
| 1 (1) | p. Cys243Tyr | 1 | 9 | M | + | + | + | - | + | - | + | - | - | - | Nephrotic syndrome | BD | Steroids, cyclosporine, MZB, colchicine, PSL |
| | | 2 | 8 | F | - | + | + | - | + | - | + | - | - | - | / | BD | PSL, steroids |
| | | 3 | 8 | F | + | + | + | - | + | - | - | - | - | - | / | BD | Glucocorticoids |
| | | 4 | 10 | F | - | + | + | - | + | - | - | - | - | - | / | BD | Colchicine |
| | | 5 | 12 | F | - | + | + | - | + | - | - | - | - | - | / | / | / |
| 2 (2) | p. Trp85Glyfs*11 | 6 | 11 | F | - | - | - | - | - | - | + | - | - | - | / | Intestinal BD | Adalimumab, PSL, MTX, NSAID, Igratimod |
| | | 7 | 0.75 | F | - | - | - | - | - | - | + | - | - | - | / | Intestinal BD | PSL |
| 3 (2) | p. His636Glufs*55 | 8 | 1 | M | - | - | - | - | + | - | - | + | - | - | Aortic valve insufficiency | Psoriatic arthritis | Infliximab, MTX, PSL, NSAIDs, colchicine |
| 4 (2) | p. Gln737Serfs*79 | 9 | 0.5 | F | - | - | - | - | + | - | - | - | - | - | BCG dermatitis | FMF | Etanercept |
| | | 10 | 5 | F | - | - | - | - | - | - | + | - | + | - | / | CD, HD | Levothyroxine |
| | | 11 | 3 | F | - | + | - | - | - | - | - | - | + | - | / | Recurrent stomatitis, HD | Levothyroxine |
| | | 12 | 3 | M | - | + | - | - | - | - | - | - | + | - | HL, craniopharyngioma | Recurrent stomatitis, HD, HL, craniopharyngioma | Levothyroxine |
| | | 13 | 1 | F | + | + | - | - | - | - | - | - | - | - | / | Recurrent fever and stomatitis | / |
| | | 14 | 0.17 | F | + | + | - | - | + | - | - | - | - | - | BCG dermatitis, aseptic meningitis | Cheilitis, BCG dermatitis, unidentified pneumonia, aseptic meningitis | Colchicine |
| 5 (2) | p. Phe637Glufs*2 | 15 | 3.5 | M | + | + | - | - | - | - | - | + | - | - | PFAPA | PFAPA | PSL, MTX, cimetidine |
| | | 16 | 3 | M | - | + | + | - | - | + | + | - | - | - | / | IgA vasculitis, recurrent stomatitis | / |
| 6 (2) | p. Cys243Tyr | 17 | 17 | M | - | + | + | - | - | - | - | - | - | - | Nephrotic syndrome | BD, nephrotic syndrome | MZB, cyclosporine A, PSL, rituximab |
| | | 18 | 10 | F | - | + | + | - | - | - | + | - | - | - | / | BD | PSL |
| | | 19 | 14 | F | - | + | + | - | - | - | - | - | - | - | / | BD | PSL |

(Continued)

| Family | Amino acid alteration | Patient | AOS (y) | Gender | RF | Oral ulcers | Genital ulcers | Ocular | Skin lesion | Vasculitis | GI ulcers | MSD | AITD | RRTI | Other manifestations | Initial diagnosis | Treatment |
|---|---|---|---|---|---|---|---|---|---|---|---|---|---|---|---|---|---|
| | | 20 | 14 | F | - | + | + | - | - | - | - | - | - | - | / | BD | PSL |
| | | 21 | 12 | F | - | + | + | - | - | - | + | - | - | - | / | BD | / |
| 7 (2) | p. Asn449Thrfs*28 | 22 | 1 | F | + | + | - | - | - | - | - | - | - | - | Pervasive developmental disorder, infectious mononucleosis | PFAPA-like recurrent fever and stomatitis, pervasive developmental disorder | Acetaminophen during fever |
| | | 23 | 3 | F | + | + | + | - | - | - | + | - | - | - | Pneumocystis carinii pneumonia | BD | Adalimumab or infliximab |
| 8 (2) | p. Ala588Valfs*80 | 24 | 0.01 | M | + | + | - | - | - | - | + | - | - | - | / | CD | Colchicine, infliximab |
| | | 25 | 20 | M | + | + | - | - | - | - | - | - | + | - | / | PFAPA-like recurrent fever and stomatitis, Graves' disease | No anti-inflammatory treatment |
| 9 (2; 3) | p. Lys417Serfs*4 | 26 | 1 | M | - | - | - | - | + | - | + | - | - | - | Autoimmune hepatitis | Autoimmune lymphoproliferative syndrome undefined | Adalimumab |
| 10 (2) | p. Arg45* | 27 | 1.08 | M | + | + | + | - | - | - | + | - | - | - | Pharyngitis | Intestinal BD | Colchicine, PSL |
| 11 (4) | p. Leu227* | 28 | 0.83 | F | + | + | + | + | - | - | + | + | - | + | Premature ovarian failure, geographic tongue, small jaw, tonsillitis | BD, JIA | MTX, thalidomide |
| | | 29 | 1.25 | F | - | + | + | + | + | - | + | + | - | - | Geographic tongue, dental crowding secondary to small jaw | BD, JIA | Infliximab |
| | | 30 | 0 | F | - | + | + | - | + | - | - | + | - | + | Goiter | BD, RA | Colchicine |
| 12 (4) | p. Phe224Serfs*4 | 31 | 10 | F | + | + | + | + | + | + | + | + | - | - | CNS vasculitis, idiopathic thrombocytopenic purpura | JIA, undifferentiated connective tissue disease, cutaneous vasculitis, SLE with CNS vasculitis | Anakinra, AZA, systemic corticosteroids |

(continued)

| Family | Amino acid alteration | Patient | AOS (y) | Gender | RF | Oral ulcers | Genital ulcers | Ocular | Skin lesion | Vasculitis | GI ulcers | MSD | AITD | RRTI | Other manifestations | Initial diagnosis | Treatment |
|---|---|---|---|---|---|---|---|---|---|---|---|---|---|---|---|---|---|
| | | 32 | 8 | F | - | + | + | - | + | - | - | + | - | - | Membranous nephropathy | BD, lupus nephritis | Anakinra |
| | | 33 | 6 | F | + | + | + | - | + | - | - | + | - | + | Lymphadenopathy, periodontal disease, intermittent arterial hypertension | Rheumatic fever, arthritis | Anakinra |
| | | 34 | 6 | F | + | + | + | - | + | - | - | + | - | + | Asthma, enamel loss, uterine fibroids | / | Anakinra |
| 13 (4) | p. Arg271* | 35 | 0.75 | M | + | + | + | - | - | - | + | + | - | + | / | BD | Colchicine |
| | | 36 | | M | - | + | + | - | - | - | - | - | - | - | / | BD | / |
| 14 (4) | p. Thr604Argfs*93 | 37 | 0.53 | F | + | + | + | - | - | - | + | - | - | + | Lymphadenopathy | PFAPA | Tofacitinib |
| 15 (4) | p. Tyr306* | 38 | 4 | F | - | + | + | - | - | - | - | - | - | + | Otitis media, recurrent urinary tract infections | BD | Infliximab |
| | | 39 | 1 | F | - | + | + | - | + | - | - | - | - | + | Dysmenorrhea, rectal bleeding, asthmatic bronchitis, rosacea | BD | Colchicine, AZA, infliximab |
| 16 (4) | p. Pro268Leufs*19 | 40 | 29 | F | + | + | + | - | + | - | - | + | + | - | / | BD | Colchicine |
| | | 41 | 15 | F | - | + | + | - | - | - | - | - | - | - | / | BD | Colchicine |
| | | 42 | 13 | F | - | + | + | - | - | - | - | - | - | - | / | BD | Colchicine |
| 17 (4) | p. Gln338* | 43 | 0.02 | M | - | + | - | - | + | + | + | - | - | - | Pulmonary embolism, CNS vasculitis, inflammatory fibroepithelial polyp | BD, CD | Systemic corticosteroids, mesalamine, dapsone, MTX, AZA, colchicine, infliximab, adalimumab, certolizumab, anakinra, canakinumab, tacrolimus, tocilizumab ineffective |
| 18 (5) | p. Trp85Glyfs*11 | 44 | 11 | M | + | + | + | + | + | - | + | + | - | - | / | Intestinal BD | Adalimumab, PSL |
| | | 45 | 0.75 | M | - | - | + | - | - | - | + | - | - | - | / | Intestinal BD | PSL |

(Continued)

| Family | Amino acid alteration | Patient | AOS (y) | Gender | RF | Oral ulcers | Genital ulcers | Ocular | Skin lesion | Vasculitis | GI ulcers | MSD | AITD | RRTI | Other manifestations | Initial diagnosis | Treatment |
|---|---|---|---|---|---|---|---|---|---|---|---|---|---|---|---|---|---|
| 19 (6) | p. Val489Alafs*7 | 46 | 10 | M | - | - | - | - | - | - | + | - | - | - | insulin-dependent diabetes, cytopenia, hepatitis, interstitial lung disease | / | PSL, sirolimus, tacrolimus, infliximab or rituximab, hematopoietic stem cell transplantation |
| 20 (7) | p. His636Terfs*1 | 47 | 16 | F | + | - | - | - | + | - | - | + | - | - | Abdominal pain | Adult-onset Stills' disease | Tocilizumab |
| | | 48 | | M | - | - | - | - | - | - | - | + | - | - | / | Early-onset RA | Colchicine |
| | | 49 | 0.67 | F | + | + | - | - | + | - | - | + | - | - | Abdominal pain | / | Tocilizumab |
| | | 50 | 5 | M | - | + | - | - | + | - | - | - | - | - | vomit | / | / |
| 21 (8) | p. Glu332* | 51 | 6 | F | - | - | - | + | - | - | + | + | - | - | Thrombophlebitis, fatigue | BD | Etanercept, colchicine |
| | | 52 | 6 | M | + | + | - | - | + | - | - | - | + | - | Vitiligo | / | Colchicine |
| | | 53 | 0.5 | F | - | - | - | - | + | - | + | + | - | - | / | BD | Colchicine, mesalazine |
| 22 (9) | p. Lys91* | 54 | 7 | F | - | - | - | - | - | - | - | - | + | - | Liver failure | Autoimmune thyroiditis | / |
| | | 55 | 7 | F | - | - | - | - | + | - | - | + | + | - | Atrophic gastritis, anemia, autoinflammatory pulmonary reaction, repeated genital HSV infections. | Autoimmune thyroiditis | / |
| | | 56 | 7 | M | - | - | - | - | - | - | - | - | + | - | / | Autoimmune thyroiditis | / |
| | | 57 | 4 | F | - | - | - | - | - | - | - | + | + | - | / | JIA | / |
| 23 (10) | p. Gln370Argfs*16 | 58 | 0.17 | F | + | + | - | - | + | - | + | + | - | - | hepatitis | CD | Thalidomide |
| 24 (10) | p. Arg439Glnfs*6 | 59 | 0.08 | M | - | + | - | - | - | - | + | + | - | - | Liver lesions and splenomegaly | JIA | Mesalazine, MTX, systemic corticosteroids, infliximab, and recombinant human TNF-II |
| 25 (10) | p. Trp85* | 60 | 0.5 | M | + | + | - | - | + | - | + | - | - | - | / | / | Mesalazine, systemic corticosteroids and thalidomide |

(Continued)

| Family | Amino acid alteration | Patient | AOS (y) | Gender | RF | Oral ulcers | Genital ulcers | Ocular | Skin lesion | Vasculitis | GI ulcers | MSD | AITD | RRTI | Other manifestations | Initial diagnosis | Treatment |
|---|---|---|---|---|---|---|---|---|---|---|---|---|---|---|---|---|---|
| 26 (11) | p. Gln187* | 61 | 7 | F | + | - | - | - | - | - | - | - | + | - | Hepatomegaly, ascites and pericardial effusion, nephritis | Liver fibrosis, hypothyroidism, SLE, lupus nephritis | Etanercept, hydroxychloroquine, PSL, mycophenolate mofetil |
| | | 62 | 2.6 | M | + | - | - | - | - | - | + | + | - | - | / | CD, RA | Etanercept, etiasa, MTX |
| | | 63 | 7 | M | - | + | - | - | - | - | + | + | - | - | Anal fistula | / | / |
| 27 (11) | p. Arg 87* | 64 | 7 | M | + | - | - | - | - | - | - | + | - | - | Interstitial lung disease | JIA, macrophage activation syndrome and interstitial lung disease | Etanercept, PSL |
| 28 (12) | p. Cys478* | 65 | 6 | M | + | + | + | - | - | - | - | - | - | - | Lymphadenopathy | BD | PSL, colchicine |
| | | 66 | 12 | F | + | + | + | - | - | - | + | - | - | - | Lymphadenopathy | BD | PSL, colchicine |
| 29 (13) | p. Asn102Ser | 67 | 19 | F | - | + | - | - | - | + | + | - | - | - | / | Intestinal BD | Glucocorticoid and thalidomide |
| | | 68 | 4 | M | + | + | - | - | + | - | - | - | - | - | / | / | No anti-inflammatory treatment |
| | | 69 | 7 | M | - | + | - | - | - | - | - | - | - | - | / | / | No anti-inflammatory treatment |
| 30 (14) | p. Arg183* | 70 | | M | - | + | - | - | - | - | - | - | - | - | / | / | / |
| | | 71 | | F | - | + | - | - | - | - | - | - | - | - | / | / | / |
| | | 72 | | F | - | + | - | - | - | - | - | - | - | - | / | / | / |
| 33 (15) | p. Lys91* | 73 | 7 | F | - | - | - | - | - | - | - | + | + | - | Hepatitis, atrophic gastritis, anemia | Autoimmune thyroid disease, cirrhosis | Thyroid replacement therapy |
| | | 74 | 4 | F | - | + | + | - | - | - | - | + | + | - | Atrophic gastritis, anemia, Hepatosteatosis | Autoimmune thyroid disease, atrophic gastritis, psoriasis, aphthous stomatitis, genital papillomatosis | Thyroid replacement therapy, etanercept, corticosteroids, econazole, mycophenolate, PSL |
| | | 75 | | M | - | - | - | - | + | - | - | - | + | - | Growth delay, atrophic gastritis, anemia | Autoimmune thyroid disease | Thyroid replacement therapy |

(Continued)

| Family | Amino acid alteration | Patient | AOS (y) | Gender | RF | Oral ulcers | Genital ulcers | Ocular | Skin lesion | Vasculitis | GI ulcers | MSD | AITD | RRTI | Other manifestations | Initial diagnosis | Treatment |
|---|---|---|---|---|---|---|---|---|---|---|---|---|---|---|---|---|---|
| | | 76 | 1.4 | F | - | - | - | - | - | - | - | + | + | - | Growth delay, cow's milk allergy | Autoimmune thyroid disease, JIA | Thyroid replacement therapy, golimumab, AZA |
| 34 (16) | Deletion of exons 2–3 | 77 | 6 | M | + | + | + | - | - | - | + | + | - | - | / | Intestinal BD | Adalimumab, MTX |
| 35 (17) | p. Arg271* | 78 | 5 | F | + | + | - | - | - | - | + | - | + | - | / | Autoimmune thyroid disease | Levothyroxine, hydroxychloroquine, thalidomide |
| | | 79 | 7 | M | + | + | + | - | + | - | + | - | + | - | / | BD, Henoch Schonlein purpura, incomplete obstruction of small intestine | / |
| 36 (17) | p. Arg45* | 80 | 0.75 | F | + | + | - | - | + | - | - | - | - | - | / | / | PSL, infliximab, sulfasalazine |
| | | 81 | 10 | F | - | + | - | - | - | - | - | - | - | - | / | BD | PSL, cyclosporine |
| 37 (18) | p. Lys303fs | 82 | 5.92 | M | - | + | - | - | + | - | + | + | - | - | / | / | Hydroxychloroquine |
| 38 (19) | p. Arg271* | 83 | 5 | F | - | + | - | + | + | + | - | - | - | - | Lupus nephritis | / | PSL, mesalazine, recombinant human TNF-II |
| 39 (19) | p. Asn98Thrfs25 | 84 | 2 | M | - | + | + | - | - | - | - | - | - | - | / | BD | Hydroxychloroquine, AZA, mycophenolate mofetil ineffective, considering IL-1 blocking therapy |
| 40 (20) | p. Cys200Alafs*16 | 85 | 14 | M | + | + | - | - | - | - | + | - | - | - | / | / | No treatment |
| | | 86 | | F | - | + | + | - | + | - | - | - | - | - | Septicemia | / | PSL |
| | | 87 | 11 | F | + | + | - | - | - | - | - | - | - | - | Abdominal pain | BD | / |
| 41 (21) | p. Met476Ile | 88 | 2 | M | + | + | - | - | + | - | + | + | - | - | Lymphadenopathy, constipation | BD | / |
| | | 89 | | F | + | + | - | - | - | - | - | - | - | - | / | / | PSL, acyclovir |

AOS: age of onset, RF: recurrent fever, GI: gastrointestinal, MSD: musculoskeletal disorder, AITD: autoimmune thyroid disorder, RRTI: recurrent respiratory tract infection, F:female, M:male, BD: Behcer’s disease, CD: crohn’s disease,

FMF: familial Mediterranean fever, BCG: bacillus Calmette–Guerin, HL: Hodgkin lymphoma, JIA: juvenile idiopathic arthritis, HD: Hashimoto disease, RA: rheumatoid arthritis, SLE: systemic lupus erythematosus, CNS: central nervous system, MZB: mizoribine, PSL: prednisolone, MTX: methotrexate, AZA: azathioprine

## References


1. Shigemura, T, Kaneko, N, Kobayashi, N, Kobayashi, K, Takeuchi, Y, Nakano, N, et al., Novel heterozygous C243Y A20/TNFAIP3 gene mutation is responsible for chronic inflammation in autosomal-dominant Behcet's disease. RMD Open. (2016) 2:e000223. doi: 10.1136/rmdopen-2015-000223

2. Kadowaki, T, Ohnishi, H, Kawamoto, N, Hori, T, Nishimura, K, Kobayashi, C, et al., Haploinsufficiency of A20 causes autoinflammatory and autoimmune disorders. J. Allergy Clin. Immunol. (2018) 141:1485-1488 e11. doi: 10.1016/j.jaci.2017.10.039

3. Takagi, M, Ogata, S, Ueno, H, Yoshida, K, Yeh, T, Hoshino, A, et al., Haploinsufficiency of TNFAIP3 (A20) by germline mutation is involved in autoimmune lymphoproliferative syndrome. J. Allergy Clin. Immunol. (2017) 139:1914-1922. doi: 10.1016/j.jaci.2016.09.038

4. Aeschlimann, FA, Batu, ED, Canna, SW, Go, E, Gul, A, Hoffmann, P, et al., A20 haploinsufficiency (HA20): clinical phenotypes and disease course of patients with a newly recognised NF-kB-mediated autoinflammatory disease. Ann. Rheum. Dis. (2018) 77:728-735. doi: 10.1136/annrheumdis-2017-212403

5. Ohnishi, H, Kawamoto, N, Seishima, M, Ohara, O, and Fukao, T, A Japanese family case with juvenile onset Behcet's disease caused by TNFAIP3 mutation. Allergol Int. (2017) 66:146-148. doi: 10.1016/j.alit.2016.06.006

6. Duncan, CJA, Dinnigan, E, Theobald, R, Grainger, A, Skelton, AJ, Hussain, R, et al., Early-onset autoimmune disease due to a heterozygous loss-of-function mutation in TNFAIP3 (A20). Ann. Rheum. Dis. (2018) 77:783-786. doi: 10.1136/annrheumdis-2016-210944

7. Lawless, D, Pathak, S, Scambler, TE, Ouboussad, L, Anwar, R, and Savic, S, A Case of Adult-Onset Still's Disease Caused by a Novel Splicing Mutation in TNFAIP3 Successfully Treated With Tocilizumab. Front. Immunol. (2018) 9:1527. doi: 10.3389/fimmu.2018.01527

8. Aeschlimann, FA, and Laxer, RM, Response to: 'A20 haploinsufficiency (HA20): clinical phenotypes and disease course of patients with a newly recognised NF-kB-mediated autoinflammatory disease' by Aeschlimann et al. Ann. Rheum. Dis. (2019) 78:e36. doi: 10.1136/annrheumdis-2018-213359

9. Rajamaki, K, Keskitalo, S, Seppanen, M, Kuismin, O, Vahasalo, P, Trotta, L, et al., Haploinsufficiency of A20 impairs protein-protein interactome and leads into caspase-8-dependent enhancement of NLRP3 inflammasome activation. RMD Open. (2018) 4:e000740. doi: 10.1136/rmdopen-2018-000740

10. Zheng, C, Huang, Y, Ye, Z, Wang, Y, Tang, Z, Lu, J, et al., Infantile Onset Intractable Inflammatory Bowel Disease Due to Novel Heterozygous Mutations in TNFAIP3 (A20). Inflamm. Bowel Dis. (2018) 24:2613-2620. doi: 10.1093/ibd/izy165

11. Li, GM, Liu, HM, Guan, WZ, Xu, H, Wu, BB, and Sun, L, Expanding the spectrum of A20 haploinsufficiency in two Chinese families: cases report. BMC Med. Genet. (2019) 20:124. doi: 10.1186/s12881-019-0856-1

12. Tsuchida, N, Kirino, Y, Soejima, Y, Onodera, M, Arai, K, Tamura, E, et al., Haploinsufficiency of A20 caused by a novel nonsense variant or entire deletion of TNFAIP3 is clinically distinct from Behcet's disease. Arthritis Res. Ther. (2019) 21:137. doi: 10.1186/s13075-019-1928-5

13. Chen, Y, Huang, H, He, Y, Chen, M, Seidler, U, Tian, D, et al., A20 Haploinsufficiency in a Chinese Patient With Intestinal Behcet's Disease-Like Symptoms: A Case Report. Front. Immunol. (2020) 11:1414. doi: 10.3389/fimmu.2020.01414

14. Liang, J, Zhang, H, Guo, Y, Yang, K, Ni, C, Yu, H, et al., Coinheritance of generalized pustular psoriasis and familial Behcet-like autoinflammatory syndrome with variants in IL36RN and TNFAIP3 in the heterozygous state. J. Dermatol. (2019) 46:907-910. doi: 10.1111/1346-8138.15034

15. Hautala, T, Vahasalo, P, Kuismin, O, Keskitalo, S, Rajamaki, K, Vaananen, A, et al., A Family With A20 Haploinsufficiency Presenting With Novel Clinical Manifestations and Challenges for Treatment. J. Clin. Rheumatol. (2020). doi: 10.1097/RHU.0000000000001268

16. Shimizu, M, Matsubayashi, T, Ohnishi, H, Nakama, M, Izawa, K, Honda, Y, et al., Haploinsufficiency of A20 with a novel mutation of deletion of exons 2-3 of TNFAIP3. Mod. Rheumatol. (2020):1-5. doi: 10.1080/14397595.2020.1719595

17. Zhong, LQ, Wang, W, Wang, L, Jiang, JJ, Shen, M, and Song, HM, [A report of clinical characteristics of 2 Chinese pedigrees with haploinsufficiency of A20 and literature review]. Zhonghua Er Ke Za Zhi. (2019) 57:922-927. doi: 10.3760/cma.j.issn.0578-1310.2019.12.006

18. Huang, YY, He, TY, Xia, Y, Luo, Y, Weng, RH, Luo, SL, et al., [Clinical phenotype and immunological features of a patient with A20 haploinsufficiency]. Zhonghua Er Ke Za Zhi. (2020) 58:218-222. doi: 10.3760/cma.j.issn.0578-1310.2020.03.011

19. Papadopoulou, C, Omoyinmi, E, Standing, A, Pain, CE, Booth, C, D'Arco, F, et al., Monogenic mimics of Behcet's disease in the young. Rheumatology (Oxford). (2019) 58:1227-1238. doi: 10.1093/rheumatology/key445

20. Sato, S, Fujita, Y, Shigemura, T, Matoba, H, Agematsu, K, Sumichika, Y, et al., Juvenile onset autoinflammatory disease due to a novel mutation in TNFAIP3 (A20). Arthritis Res. Ther. (2018) 20:274. doi: 10.1186/s13075-018-1766-x
21. Dong, X, Liu, L, Wang, Y, Yang, X, Wang, W, Lin, L, et al., Novel Heterogeneous Mutation of TNFAIP3 in a Chinese Patient with Behcet-Like Phenotype and Persistent EBV Viremia. J. Clin. Immunol. (2019) 39:188-194. doi: 10.1007/s10875-019-00604-9
