# Supplementary material for: Association of Clinical Phenotypes in Haploinsufficiency A20 (HA20) With Disrupted Domains of A20
Source: Front Immunol. 2020 Sep 23;11:574992. doi: 10.3389/fimmu.2020.574992 (PMC7546856; doi:10.3389/fimmu.2020.574992)
Supplement: Supplementary file 3 [file Data_Sheet_3.PDF]

Supplementary Material

Table S2. Univariate analysis of organ involvement in patients with HA20 based on TNFAIP3 mutation loci.

Mutation loci	Total (n=89)	Mild (n=50)	Moderate (n=30)	Severe (n=9)	Fisher	P value
**p.Cys243Tyr**	11%(10/89)	16%(8/50)	7%(2/30)	0%(0/9)	2.074	0.313
**p.Trp85Glyfs*11**	4%(4/89)	6%(3/50)	3%(1/30)	0%(0/9)	0.446	1.000
**p.Trp85***	1%(1/89)	2%(1/50)	0%(0/30)	0%(0/9)	1.456	1.000
**p.His636Glufs*55**	1%(1/89)	2%(1/50)	30%(0/30)	0%(0/9)	1.456	1.000
**p.His636Terfs*1**	4%(4/89)	6%(3/50)	3%(1/30)	0%(0/9)	0.446	1.000
**p.Gln737Serfs*79**	7%(6/89)	10%(5/50)	3%(1/30)	0%(0/9)	1.262	0.559
**p.Phe637Glufs*2**	2%(2/89)	2%(1/50)	3%(1/30)	0%(0/9)	0.858	1.000
**p.Asn449Thrfs*28**	2%(2/89)	2%(1/50)	3%(1/30)	0%(0/9)	0.858	1.000
**p.Ala588Valfs*80**	2%(2/89)	4%(2/50)	0%(0/30)	0%(0/9)	1.263	0.617
**p.Lys417Serfs*4**	1%(1/89)	2%(1/50)	0%(0/30)	0%(0/9)	1.456	1.000
**p.Arg45***	3%(3/89)	4%(2/50)	3%(1/30)	0%(0/9)	0.408	1.000
**p.Leu227***	3%(3/89)	0%(0/50)	3%(1/30)	22%2/9)	7.462	0.010
						0.375[a]
						0.021[b]
						0.127[c]
**p.Phe224Serfs*4**	4%(4/89)	0%(0/50)	3%(1/30)	33%(3/9)	11.351	0.001
						0.375[a]
						0.003[b]
						0.032[c]
**p. Arg271***	6%(5/89)	4%(2/50)	10%(3/30)	0%(0/9)	1.420	0.501
**p.Thr604Argfs*93**	1%(1/89)	0%(0/50)	3%(1/30)	0%(0/9)	2.477	0.438
**p.Tyr306***	2%(2/89)	0%(0/50)	3%(1/30)	11%(1/9)	4.288	0.078
**p.Pro268Leufs*19**	3%(3/89)	4%(2/50)	3%(1/30)	0%(0/9)	0.408	1.000
**p.Gln338***	1%(1/89)	0%(0/50)	0%(0/30)	11%(1/9)	4.885	0.101
**p.Val489Alafs*7**	1%(1/89)	0%(0/50)	3%(1/30)	0%(0/9)	2.477	0.438
**p.Glu332***	3%(3/89)	2%(1/50)	7%(2/30)	0%(0/9)	1.457	0.676
**p.Lys91***	4%(4/89)	6%(3/50)	0%(0/30)	11%(1/9)	2.854	0.148
**p.Gln370Argfs*16**	6%(5/89)	0%(0/50)	13%(4/30)	11%(1/9)	7.248	0.020
						0.017[a]
						0.153[b]
						0.560[c]
**p.Arg439Glnfs*6**	1%(1/89)	2%(0/50)	3%(1/30)	0%(0/9)	2.477	0.438
**p.Gln187***	3%(3/89)	6%(2/50)	3%(1/30)	0%(0/9)	0.408	1.000
**p.Arg87***	1%(1/89)	2%(1/50)	0%(0/30)	0%(0/9)	1.456	1.000
**p.Cys478***	2%(2/89)	4%(1/50)	3%(1/30)	0%(0/9)	0.858	1.000
**p.Asn102Ser**	3%(3/89)	6%(3/50)	0%(0/30)	0%(0/9)	1.665	0.485

(Continued)

Mutation loci	Total (n=89)	Mild (n=50)	Moderate (n=30)	Severe (n=9)	Fisher	P value
**p.Arg183***	3%(3/89)	6%(3/50)	0%(0/30)	0%(0/9)	1.665	0.485
**Deletion of exons 2–3**	1%(1/89)	2%(0/50)	3%(1/30)	0%(0/9)	2.477	0.438
**p.Lys303fs**	1%(1/89)	2%(0/50)	3%(1/30)	0%(0/9)	2.477	0.438
**p.Asn98Thrfs*25**	1%(1/89)	2%(1/50)	0%(0/30)	0%(0/9)	1.456	1.000
**p.Cys200Alafs*16**	3%(3/89)	6%(2/50)	3%(1/30)	0%(0/9)	0.408	1.000
**p.Met476Ile**	2%(2/89)	4%(1/50)	3%(1/30)	0%(0/9)	0.858	1.000

The disease degree of HA20 is classified into three levels based on the number of involved organs. Mild: less than 4 organs involved, Moderate: 4 to 6 organs involved, Severe: more than 6 organs involved.

[a]Comparison of organ involvement between OTU+ZnF and OTU groups; [b]Comparison of organ involvement between OTU+ZnF and ZnF groups; [c]Comparison of organ involvement between OTU and ZnF groups.
